# Tracking of serum lipids in healthy children on a year-to-year basis

**DOI:** 10.1186/s12872-023-03391-9

**Published:** 2023-08-02

**Authors:** Ludwig Maidowski, Wieland Kiess, Ronny Baber, Anne Dathan-Stumpf, Uta Ceglarek, Mandy Vogel

**Affiliations:** 1 University of Leipzig, LIFE Child Leipzig Research Center for Civilization Diseases, Philipp-Rosenthal-Str. 27, 04103 Leipzig, Germany; 2 University of Leipzig, Hospital for Children and Adolescents, Liebigstr. 20a, 04103 Leipzig, Germany; 3 University of Leipzig, Institute of Laboratory Medicine, Clinical Chemistry and Molecular Diagnostics, Paul-List-Str. 13-15, 04013 Leipzig, Germany; 4 University of Leipzig, Leipzig Medical Biobank, Liebigstr. 27, 04103 Leipzig, Germany; 5 University of Leipzig, Department of Women and Child Health, Liebigstr. 20a, 04103 Leipzig, Germany

**Keywords:** Tracking, Lipids, Children, Lipid profiles

## Abstract

**Objectives:**

To assess the stability of lipid profiles throughout childhood and evaluate their onset and dynamic.

**Materials and methods:**

Lipid markers were longitudinally measured in more than 1300 healthy children from the LIFE Child study (Germany) and categorized into *normal*, *at-risk*, or *adverse*. Year-to-year intra-person persistence of the categories during follow-ups was examined and Pearson’s correlation coefficient was calculated.

**Results:**

We found strong positive correlations for TC, LDL-C and ApoB (*r* > 0.75, *p* < 0.001) from the age of four years. Correlations were lowest during the first two years of life. Most children with *normal* levels also had *normal* levels the following year. Children with *at-risk* levels showed a tendency towards *normal* levels at the follow-up visit. *Adverse* levels of TC, LDL-C, ApoB (all ages), and HDL-C (from age 15) persisted in more than half of the affected children. Age-dependent patterns of stability were most pronounced and similar for TC, LDL-C, and ApoB.

**Conclusions:**

*Normal* levels of serum lipids show high stability and *adverse* levels stabilized in early childhood for TC, LDL, and ApoB. *At-risk* and *adverse* levels of TC, LDL-C or ApoB may warrant further or repeated diagnostic measurements with regards to preventing CVD in the long run.

**Supplementary Information:**

The online version contains supplementary material available at 10.1186/s12872-023-03391-9.

## Introduction

Cardiovascular diseases (CVD) are the leading cause of death globally [1] and in Germany [2, 3]. Known risk factors include unfavorable lipid profiles [4, 5], which have often been present for years before the actual diagnosis of CVDs [4, 6]. Whilst unfavorable levels of total cholesterol (TC), low- and high-density lipoprotein cholesterol (LDL-C, HDL-C), triglycerides (TG), and apolipoproteins A1 and B (apolipoprotein A, apolipoprotein B) are related to distinct BMI trajectories [7–16] and weight status [17–22], they may promote the pathogenesis of atherosclerosis [23] independent of BMI [24, 25].

Therefore, monitoring blood lipid levels could help prevent the development of CVD by identifying susceptible individuals as early as possible. Studies from various countries such as the US [26], China [27], Iran [28] or Northern Europe [29, 30] have reported the influence of sex, age and ethnicity on serum lipids. In Germany, percentile curves for serum lipids based on large cohort studies of healthy children showed considerably varying levels throughout childhood. Higher levels were seen during the first three years and following puberty. Girls tended to have higher levels than boys [31–33]. The prevalence of dyslipidemia in Germany was reported to be similar to the prevalence in the United States [31]. In light of this, the transition to, respective the persistence of, (non-)pathological serum lipid status during childhood, adolescence, and early adulthood is of interest as it may indicate increased risk for later CVD. Establishing better understanding of the dynamics of lipid profiles throughout childhood is important when considering the implementation of screening protocols for susceptible individuals.

Longitudinal studies such as the Bogalusa heart study [34–36], the Project Heartbeat! [37, 38] and others [26, 39–42] indicate that unfavorable lipid profiles are likely to persist during childhood into adulthood. However, studies often comprised only two waves of data collection [34, 35, 40, 41]. Thus, they fail to identify the periods when serum lipid levels find their future, stable level. Moreover, infancy and early childhood are rarely examined. One study reported lipid levels from six months to four years of age but did not include further follow-up measurements [43]. Two studies analyzed lipid levels in cord blood, with the first follow-up measurement not before the age of six [44, 45]. Another study investigated children aged 4–18 years at baseline but included only one follow-up (four years later) [34]. Yet another study followed children at 4-month intervals for four years but did not include children younger than eight years [37, 38]. Similarly, some other study could show tracking and age-dependency for children aged 11–14 years [46]. Therefore, year-to-year intra-person data may complement those studies by identifying periods during which lipid levels stabilize. To our knowledge, there is no study covering the entire age range from three months to 18 years of age.

This study aimed to examine lipid profile trajectories between 0.25 and 18 years of age. We addressed the following questions: Are serum lipid levels (TC, LDL-C, HDL-C, TG, apolipoprotein A, apolipoprotein B) stable throughout childhood? Does the stability depend on age? Are there differences between those laboratory parameters?

## Methods

### Study population

The current study is part of the LIFE Child study. LIFE Child study is an ongoing longitudinal and observational study program based in Leipzig (Germany) which assesses the development of healthy children aged between 0 and 18 years with annual follow-up visits. It consists of three cohorts. The LIFE Child Birth cohort includes children up to age one. The LIFE Child Health cohort includes children between the ages of one and 20 with a variable age of initial participation. Children from the LIFE Child Birth cohort are usually integrated in the LIFE Child Health cohort. The LIFE Child Obesity cohort includes children between the ages of six and 20 years with a BMI > 97th percentile of German age- and sex-specific norms according to the guidelines of the German Working Group on Childhood and Adolescent Obesity (AGA) of the German Obesity Society (DAG) and the German Society of Pediatrics and Adolescent Medicine (DGKJ) [47]. Study participants are recruited from the general public or the University hospital Leipzig. Children with syndromic disorders are excluded [48]. Thus, except for the LIFE Child Obesity cohort, study participants are generally assumed to be healthy. The study was approved by the Ethical Committee (Institutional Review Board [IRB]) of the Medical Faculty, University of Leipzig (reg. no. 477–19-ek-03122020). Informed consent to participate was obtained from all subjects and/or their legal guardian(s). More information on the LIFE Child study, the recruitment of study participants, the study population and the assessments carried out during each visit are described in detail elsewhere [48, 49]. The current study used anthropometry measurements as well as the assessment of blood samples from children of all three cohorts recruited between 2011 and 2020. In a first step, children were excluded based on laboratory measurements as in particular impaired liver and/or renal function would influence serum lipids. Thus, children with pathological levels of alanine aminotransferase (ALT), aspartate aminotransferase (AST), HbA1c, creatinine, gamma glutamyl transpeptidase (GGT) or thyroid-stimulating hormone (TSH) were excluded. In a second step, children with no follow-up visits were excluded.

### Lipid measurements

Age- and weight-adapted volumes of venous blood were taken in the morning. From the age of seven, children were asked to fast eight hours. In case of non-adherence to adequate fasting times, this was documented using standardized questionnaires [49]. Blood samples have been processed at the Leipzig Medical Biobank following standardized operating procedures. Serum lipids were measured using a Cobas 8000 Clinical Chemistry Analyzer (Roche Diagnostics GmbH, Mannheim, Germany; 1st gen. used until 18.03.2016, 2nd gen. afterwards) at the Institute for Laboratory Medicine of the University of Leipzig. TC, HDL-C, LDL-C, TG were determined using a validated specific homozygous enzymatic color test. Apolipoprotein A and apolipoprotein B were determined by an immunological turbidimetric test.

### Statistical analysis

From the age group 2 upwards, age was categorized as rounded chronological age (e.g., age group 2 consists of children aged 1.5 to < 2.5 years of age and so forth up to age group 18 (17.5 to < 18.5 years of age). Because during the first year of life, there were three planned visits at three, six and twelve months of age, those were defined as three additional age groups (age groups 3 M, 6 M and 1) We converted TC, LDL-C, HDL-C, TG, apolipoprotein A, and apolipoprotein B to standard deviation scores (SDS), using previously published references based on the entire LIFE Child cohort [31]. Lipid profiles were categorized based on age- and sex-specific SDS. Comparable to other studies, SDS < 0.67 (75^th^ percentile) (or > -0.67 (25^th^ percentile) for HDL-C, apolipoprotein A) were referred to as *normal* levels of serum lipids. For unfavorable levels, studies have used different cut-off values between 0.67 and 1.28 SDS (75^th^ and 90^th^ percentile) [5, 34, 35]. According to a German guideline for diagnosis and treatment of hyperlipidemia in children and adolescence from 2015, serum lipids roughly equivalent to > 75th percentile are described as borderline and serum lipids roughly equivalent to > 95th percentile as high [50]. Generally, no apparent consensus regarding which SD scores should be used seems to exist. Reflecting that overweight is usually defined as BMI > 90^th^ percentile [51] and in accordance with previous research, unfavorable lipid profiles were defined in our study as age- and sex-specific SDS ≥ 1.28 (90^th^ percentile, for TC, LDL-C, TG, and apolipoprotein B) and ≤ -1.28 (10^th^ percentile, for HDL-C and apolipoprotein A), respectively [8, 21, 52]. We refer to these as *adverse* levels of serum lipids. Neither *normal* nor *adverse* SDS (i.e., 0.67 to 1.28 SDS or, in the case of HDL-C and apolipoprotein A -1.28 to -0.67 SDS) are referred to as *at-risk* levels of serum lipids.

Descriptive statistics were given as means and standard deviation for all continuous and counts and percentages for all categorical variables. We calculated the Pearson’s product-moment correlation (r) for SDS values of TC, LDL-C, HDL-C, TG, apolipoprotein A, and apolipoprotein B for each pair of consecutive visits. Further, we estimated the likelihood of belonging to one of the groups *normal, at risk,* or *adverse* stratified by the group membership the year before as prospective relative frequencies (pRF) (e.g., the likelihood of being assigned to the group with adverse levels of TC at age five when being in that same group at age four). For visualization of trends, pRF were modeled dependent on age using locally weighted regression. A sensitivity analysis regarding the effects of including the LIFE Child Obesity cohort was carried out. The preparation and analysis of the data were carried out with the free statistical software R version 4.0.5 [53].

## Results

### Population characteristics

The initial dataset consisted of 16927 measurements from 4653 children. Of those, 1313 children aged three months to 18 years (652 female) had at least two data points for one or more of the parameters TC, LDL-C, HDL-C, TG, apolipoprotein A or apolipoprotein B. Finally, 3809 measurements were included. The median number of measurements per child was three, the maximum number was nine. On average, 103 children per age group were included (min. *n* = 28, max. *n* = 221). The data was equally distributed across both sexes (50.3% male children). The mean BMI SDS was 0.12 with female presenting a higher mean than male children (0.14 vs 0.10). Girls entered puberty, based on Tanner stages, earlier than boys. *Supplement *1 gives an overview of clinical and sociodemographic characteristics of the study population.

### Levels of serum lipids

In Table 1, mean and standard deviation for all laboratory parameters are presented stratified by group (*normal*, *at-risk* or *adverse*). For all parameters and age groups, approximately 75% of the measurements were categorized as *normal*. Approximately 15%/10% were categorized as *at-risk*/*adverse*, respectively. Supplement 2 shows the mean lipid levels stratified by age.

**Table 1:** Mean levels of serum lipids (TC, LDL, HDL, TG, ApoA, ApoB) by sex and group

	**all**					**male**					**female**				
	**all**	**normal**	**at-risk**	**adverse**	*p*.overall*	**all**	**normal**	**at-risk**	**adverse**	*p*.overall*	**all**	**normal**	**at-risk**	**adverse**	*p*.overall*
TC (mmol/l)	4.12 (0.74)	3.82 (0.50)	4.81 (0.22)	5.52 (0.48)	0.000	4.18 (0.74)	3.88 (0.50)	4.93 (0.13)	5.63 (0.46)	0.000	4.07 (0.73)	3.75 (0.50)	4.72 (0.23)	5.41 (0.47)	0.000
	*N* = *3567*	*N* = *2717 (76.17%)*	*N* = *507 (14.21%)*	*N* = *343 (9.62%)*		*N* = *1798*	*N* = *1407 (78.25%)*	*N* = *224 (12.46%)*	*N* = *167 (9.29%)*		*N* = *1769*	*N* = *1310 (74.05%)*	*N* = *283 (16.00%)*	*N* = *176 (9.95%)*	
LDL (mmol/l)	2.36 (0.64)	2.10 (0.42)	2.94 (0.16)	3.57 (0.41)	0.000	2.42 (0.64)	2.15 (0.41)	3.03 (0.12)	3.68 (0.39)	0.000	2.31 (0.63)	2.04 (0.41)	2.87 (0.16)	3.46 (0.41)	0.000
	*N* = *3595*	*N* = *2732 (75.99%)*	*N* = *493 (13.71%)*	*N* = *370 (10.29%)*		*N* = *1811*	*N* = *1402 (77.41%)*	*N* = *228 (12.59%)*	*N* = *181 (9.99%)*		*N* = *1784*	*N* = *1330 (74.55%)*	*N* = *265 (14.85%)*	*N* = *189 (10.59%)*	
HDL (mmol/l)	1.50 (0.39)	0.96 (0.17)	1.17 (0.16)	1.64 (0.34)	0.000	1.49 (0.37)	0.97 (0.16)	1.17 (0.14)	1.63 (0.31)	< 0.001	1.52 (0.41)	0.95 (0.18)	1.17 (0.18)	1.66 (0.36)	< 0.001
	*N* = *3557*	*N* = *2661 (74.81%)*	*N* = *532 (14.96%)*	*N* = *364 (10.23%)*		*N* = *1793*	*N* = *1325 (73.90%)*	*N* = *284 (15.84%)*	*N* = *184 (10.26%)*		*N* = *1764*	*N* = *1336 (75.74%)*	*N* = *248 (14.06%)*	*N* = *180 (10.20%)*	
TG (mmol/l)	0.94 (0.67)	0.71 (0.33)	1.33 (0.53)	2.21 (1.08)	0.000	0.98 (0.66)	0.75 (0.32)	1.37 (0.50)	2.20 (1.06)	< 0.001	0.91 (0.68)	0.67 (0.33)	1.28 (0.56)	2.23 (1.10)	< 0.001
	*N* = *3528*	*N* = *2686 (76.13%)*	*N* = *498 (14.12%)*	*N* = *344 (9.75%)*		*N* = *1783*	*N* = *1365 (76.56%)*	*N* = *236 (13.24%)*	*N* = *182 (10.21%)*		*N* = *1745*	*N* = *1321 (75.70%)*	*N* = *262 (15.01%)*	*N* = *162 (9.28%)*	
ApoA (g/l)	1.43 (0.23)	1.07 (0.14)	1.23 (0.08)	1.52 (0.19)	0.000	1.43 (0.23)	1.07 (0.15)	1.24 (0.07)	1.52 (0.19)	< 0.001	1.43 (0.24)	1.07 (0.13)	1.23 (0.10)	1.51 (0.20)	< 0.001
	*N* = *3601*	*N* = *2689* (74.67%)	*N* = *590 (16.38%)*	*N* = *322 (8.94%)*		*N* = *1808*	*N* = *1338 (74.00%)*	*N* = *290 (16.04%)*	*N* = *180 (9.96%)*		*N* = *1793*	*N* = *1351 (75.35%)*	*N* = *300 (16.73%)*	*N* = *142 (7.92%)*	
ApoB (g/l)	0.75 (0.18)	0.67 (0.12)	0.91 (0.05)	1.08 (0.11)	0.000	0.77 (0.19)	0.68 (0.12)	0.93 (0.05)	1.12 (0.12)	0.000	0.73 (0.18)	0.65 (0.12)	0.88 (0.04)	1.05 (0.10)	0.000
	*N* = *3618*	*N* = *2664 (73.63%)*	*N* = *565 (15.62%)*	*N* = *389 (10.75%)*		*N* = *1816*	*N* = *1345 (74.06%)*	*N* = *290 (15.97%)*	*N* = *181 (9.97%)*		*N* = *1802*	*N* = *1319 (73.20%)*	*N* = *275 (15.26%)*	*N* = *208 (11.54%)*	

*n* number of observations

^*^p-values shown refer to an analysis of variance. In addition, post-hoc test revealed significant differences for all possible 2-group comparisons

### Correlations between subsequent measurements

**Fig. 1:**
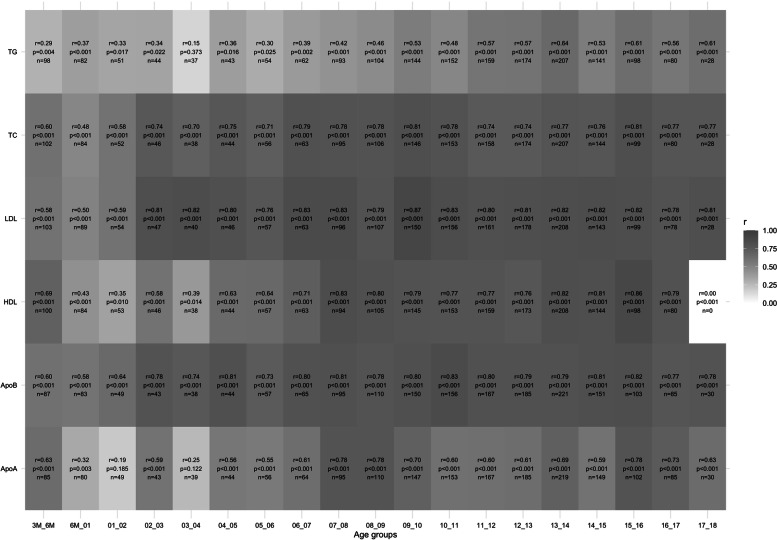
Pearson’s product moment correlation coefficient by age group; n = number of children included

We found strong positive correlations for LDL-C (0.76 ≤ r ≤ 0.87), apolipoprotein B (0.73 ≤ r ≤ 0.83), and TC (0.71 ≤ r ≤ 0.81) from the age of four years. For HDL-C and apolipoprotein A, the correlations were slightly lower and varied between *r* = 0.63 and *r* = 0.86 (HDL-C) and *r* = 0.56 and *r* = 0.78 (apolipoprotein A), respectively. TG reached comparable stability only from the age of nine years (0.53 ≤ r ≤ 0.64). In general, correlations were lowest and least stable during the first three years of life, with correlations between *r* = 0.35 and *r* = 0.82 for HDL-C, LDL-C, apolipoprotein B, and TC. For apolipoprotein A and TG, r reached values of approximately 0.3 during the same age range. Only the correlations between the measurements at three and six months and two and three years of age were slightly higher, in the case of the former, most likely due to the shorter time gap between them. The correlations are shown in detail in Fig. 1.

### Risk profile trajectories

Most children with *normal* levels also had *normal* levels the following year. Again, this was especially true from the age of four, with nearly all pRFs ≥ 80%. But even before, none of the pRFs was below 70%. With values of approximately 90%, the pRFs were particularly high for TC, LDL-C, and apolipoprotein B (from the age of four), which is consistent with the high correlations described above. In addition, children with *normal* levels of serum lipids were unlikely to develop *adverse* or *at-risk* levels the following year (Supplement 3–8 and Additional file 2: Figs. 4-9).

In general, for children in the *at-risk* group, having *normal* values the subsequent year was approximately as likely as having *at-risk* or *adverse* levels combined. The pRFs for *normal* levels the following year were lowest for apolipoprotein B (36% ≤ pRF ≤ 65%, with increasing percentages with increasing age), reflecting its stable pattern. Hence, one half up to two-thirds of children having *at-risk* apolipoprotein B-values had also *at risk* values the following year. For TC and LDL-C, between 44 and 59% of the *at-risk* group switched back to *normal* the following year. The rates did not show a clear age trend. The percentage of persistent *at-risk* values was lowest in TG between three and seven years of age (11% ≤ pRF ≤ 20%); i.e., most of these children switched back to *normal* within one year (Supplement 3–8 and Additional file 2: Figs. 4-9).

**Fig. 2:**
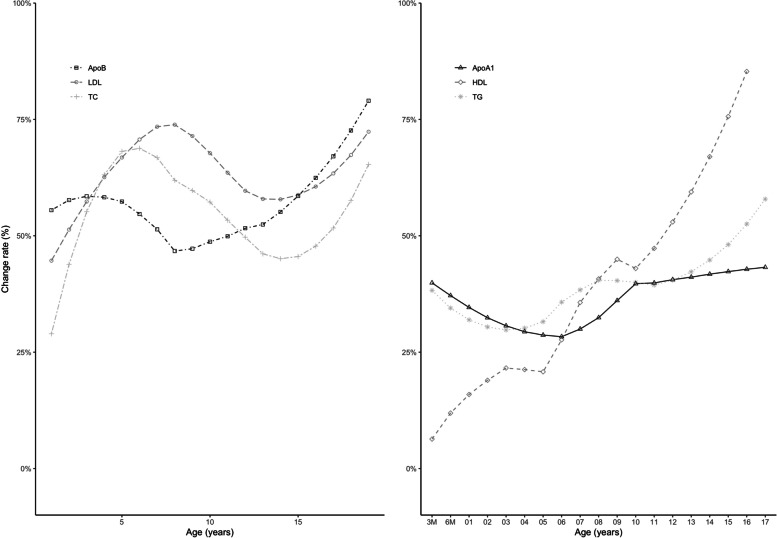
Relative frequencies of children with persistent *adverse* serum levels for TC, LDL-C, apolipoprotein B (left side) and TG, HDL-C, apolipoprotein A (right side)

Concerning children with *adverse* serum lipid levels, we found two distinct groups of lipids: apolipoprotein B, LDL-C, and TC showed relatively stable percentages of persistent *adverse* levels from the age of one year, highest for LDL-C (57% ≤ pRF ≤ 74%). For apolipoprotein B and TC, the percentages varied between 45 and 79%. Between the 3-months and the 6-months measurement, the rates of persistent *adverse* values were lowest. For HDL-C and TG, the rates of persistent *adverse* values were low for younger ages but increased with increasing age. The trend was most pronounced for HDL-C, where pRF was below 20% during the first two years of life but reached rates > 50% from the age of 12 years. There was no clear trend for apolipoprotein A, with rates varying between 28 and 43%. Figure 2 shows the pRF trajectories for children with *adverse* levels of serum lipids in two consecutive years.

## Discussion

Tracking unfavorable serum lipid levels has been used before to examine whether or not they remain stable over time [41]. Earlier studies have described the respective trajectories from childhood to young adolescence [26, 34, 35, 40, 44], as well as into adulthood [34, 35, 54]. Based on 3809 lipid measurements from 1313 children, we analyzed year-to-year changes throughout childhood (three months to 18 years of age).

### Tracking of serum lipids

In line with the Bogalusa Heart Study [34] and the Pune Children’s study [40], our study showed differences in the age-dependent stability of lipid levels between children of a German cohort with *normal* and *adverse* levels of serum lipids. In addition, our findings imply that children with *at-risk* serum lipid levels should be monitored as the levels tend to show high variability with a certain risk of switching to *adverse* levels.

**Fig. 3:**
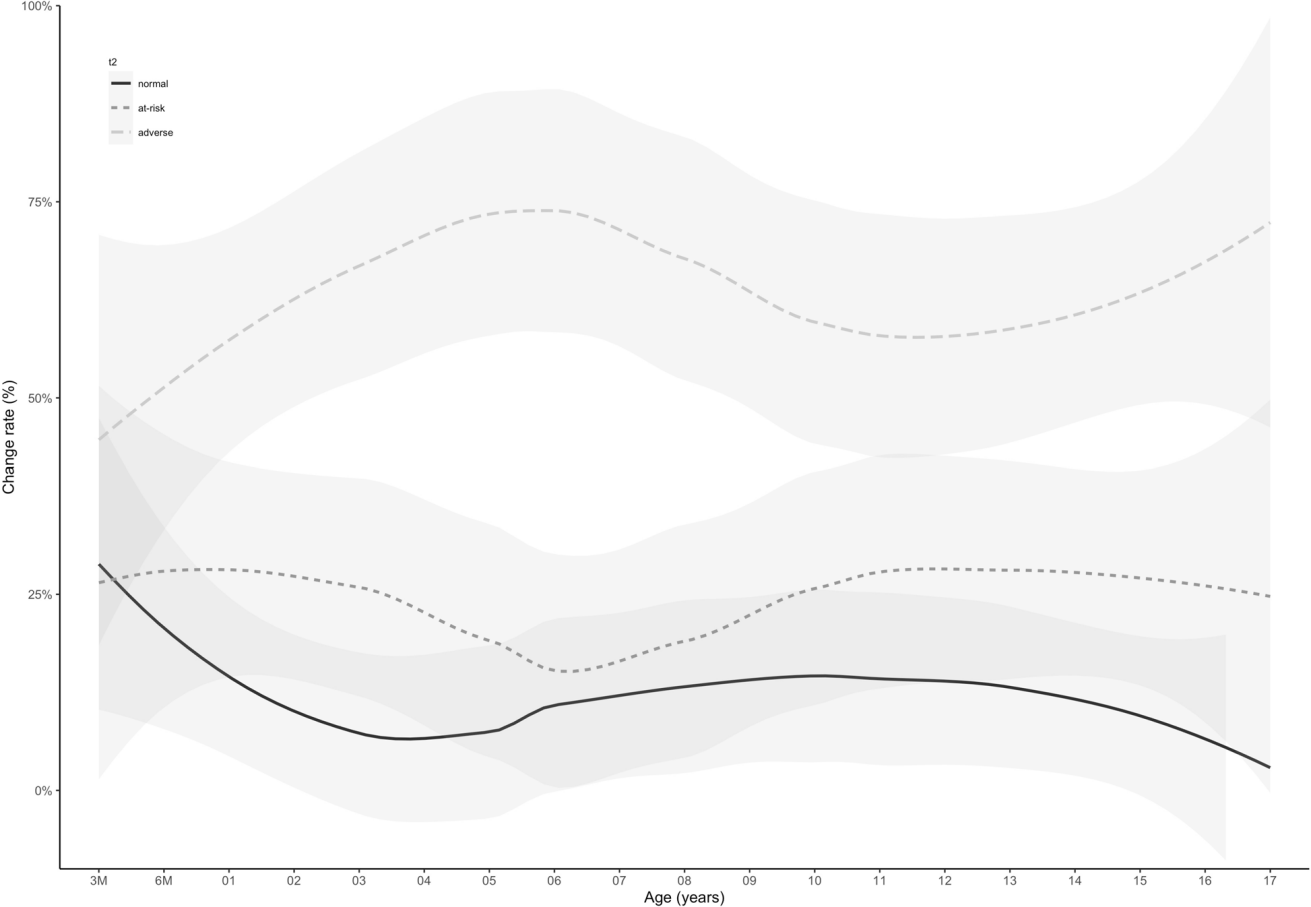
Mean relative frequencies of children with *adverse* serum levels of LDL-C at t1 and either *normal*, *at-risk* or *adverse* serum levels of LDL-C at t2

Children with *normal* serum lipid levels showed high stability for all parameters irrespective of the children’s age. Studies investigating in particular low or normal serum lipid levels reported similar findings [34, 40, 55]. Notably, in children with *adverse* levels of TC, LDL-C or apolipoprotein B, comparable stability was observed (s. Fig. 2 and for LDL-C Fig. 3). This aligns with other studies [41], which also found higher stability for TC [42] and LDL-C [34, 39–41, 56] than for TG and HDL-C [55]. As our study population also comprised an obese subcohort (LIFE Child Obesity), we repeated our analysis for TC, LDL-C and apolipoprotein B on the normal-weight subcohort. No differences in Pearson’s product-moment correlation and rPF were found, indicating that our findings were not sensitive to the inclusion of obese subjects (Supplement 9).

Children with *at-risk* levels of serum lipids were—in general—as likely to show *normal* levels as they were to show *at-risk* and *adverse* levels combined the following year. Another study also reported a tendency towards normalized levels, most likely because of regression to the mean [36]. However, a comparison is generally difficult, as different cut-off values (75^th^ or 80^th^ percentile) were used to define high-risk populations without differentiation between *at-risk* and *adverse* lipid profiles [5, 34, 35]. A more granular categorization of the former group into multiple subgroups might allow for better analysis of the variability shown in our study and an estimation which, if at all, cut-off values should be used to differentiate *normal* and *adverse* from *at-risk* lipid profiles. This would require sufficient data per group which was not given in our study. Nonetheless, our findings suggest that combining the *at-risk* and *adverse* groups into a single group might overestimate the magnitude of persistence in the risk category as only *adverse* levels showed high stability. Still, the high variability of the *at-risk* values compared to *normal* or *adverse* levels might warrant close monitoring, nonetheless.

### Age group dependent changes

Most studies on serum level trajectories are based on time intervals considerably larger than our year-to-year approach. This makes it difficult to identify critical age spans in childhood development. Our data shows that the variability varies with age. Therefore, a child’s age is important when in assessing the predictive value of lipid profiles.

First, *adverse* lipid profiles are more stable at a higher age. This was particularly apparent for TC, LDL-C and apolipoprotein B and has been reported before [34, 35, 39]. The steep increase in pRF for children with *adverse* levels of HDL-C in our study is consistent with, e.g., *Nicklas *et al*.*, who reported HDL-C levels to be relatively stable. Yet, because they included children aged five to 14 years at baseline, they were not able to grasp the dynamic changes we described.

Second, our findings are in line with several studies showing that puberty was associated with lower levels of TC and LDL-C [26, 37, 38, 57]. Age-dependent percentile curves show a similar pattern (pre-pubertal increase, decrease during puberty and post-pubertal increase) [31–33]. Studies have proposed that this fluctuation cannot be fully explained by lifestyle-related changes such as smoking or by changes in body mass index [33]. Rather, pubertal changes and related varying levels of hormones such as growth hormone or testosterone have been proposed to promote changes in serum lipids [38, 58]. Studies have also suggested the importance of family history [59] and genetics [56]. Consequently, chronological age was not seen as reliable indicator for serum lipids during puberty [33]. Interestingly, the trends of relative frequencies mirror this development. This is particularly true for *adverse* levels of TC (Supplememt 3, Additional file 2: Fig. 4) and, less pronounced, for LDL-C and apolipoprotein B. The important question of whether pre-pubertal *adverse* or *at-risk* levels track beyond puberty can only indirectly be addressed by our year-to-year approach. However, studies have shown relatively stationary measurements from pre- and post-puberty to adulthood [26, 34, 35]. Thus, the patterns seen in our study should not be necessarily interpreted as an indication that *adverse* levels of TC or LDL-C are only relevant when occurring after puberty. Yet, the increased fluctuation highlights the need for cautious interpretation of lipid profiles during puberty. Additional studies examining tracking from pre- to post-puberty are needed.

This is particularly important as we found that TC, LDL-C and apolipoprotein B start to level off earlier than HDL-C and TG. Moreover, children with *adverse* levels of TC, LDL-C and apolipoprotein B show a high degree of stability in early childhood, even at two or three years. Such early peak stability of *adverse* serum lipids levels is all the more worth mentioning because most studies lack the respective data from infancy and early childhood [44], only including children aged five years or older [26, 35, 40]. Furthermore, the results are in line with patterns of BMI changes in early childhood as reported in previous studies. In particular, an early BMI increase was indicative of both high BMI in late adolescence [60] and increased TC [22], non-HDL, apolipoprotein B and apolipoprotein B/A1-ratio [10]. However, it is important to note that a sensitivity analysis showed that our main findings concerning TC, LDL-C and apolipoprotein B were insensitive to the inclusion of the LIFE Child Obesity cohort, highlighting the fact that unfavorable lipid profiles may exists before the definition of obesity is met. Therefore, the described stability of *adverse* lipid profiles warrants serious attention even at such a young age. As our results suggest, this is particularly true for TC, LDL-C and apolipoprotein B.

Our findings provide additional data supporting a differentiated approach to screening for cardiovascular risk factors in healthy children. Not all serum lipids are equally suited as only TC, LDL-C and apolipoprotein B showed considerable stability. This is important as especially LDL-C is used for defining treatment goals in clinical settings [61]. The fact that *adverse* lipid profiles tend to be stable throughout childhood and adolescence suggests that children should be tested for dyslipidemia in early childhood to identify those with already elevated serum lipids. However, cautious interpretation of such results is needed as serum lipids are known to be influenced by various factors such as age, sex, body composition and genetics. This highlights the importance of an individual approach when assessing a child’s lipid profile at young age. Possible diagnostic and therapeutic consequences need to be considered. Before proposing additional measures at such a young age a thorough risk–benefit analysis which adequately reflects the risk of attributing pathological laboratory measurements to young children and as well as acting on the basis of possible false-positive results has to take place. In all cases, the age of the child, its current weight status [22], known risk factors for familial hypercholesterolemia and familial history of cardiovascular events need to inform the decision making process. This said, our study implies differing follow-up strategies after an initial blood sample. As a rule of thumb, our study suggests that children with *normal* serum lipids levels might not profit from another test unless indicated for other reasons. Children with *at-risk* serum lipids levels might profit from another measurement post-puberty. Children with *adverse* serum lipids levels should be assessed based on their individual risk profile. If no additional cardiovascular risk factors are present, another measurement post-puberty might suffice. Those with known additional risk factors should be monitored more closely. In any case, general recommendations regarding eating habits, exercise and aiming at normal weight should be given [62]. Children with *at-risk* serum lipids levels might profit from another measurement post-puberty.

### Strengths and weaknesses

Our study could analyze 3809 measurements from more than 1300 children, covering the age from three months to 18 years. This allowed for the analysis of dynamic changes during childhood, thereby complementing already existing research based on larger age spans. Standardized procedures (standardized time of blood withdrawal, standardized processing, and analysis protocols) were carried out by trained professionals. Sample processing and analysis were performed by the same institute. Whilst participants of the LIFE Child study often take part in multiple follow-ups, gaps of sometimes several years between those assessments are not uncommon. Thus, not all children included had measurements for all age groups. Due to our requirement of two consecutive measurements, the number of children included in our study varied for different age groups. Especially for the age from two to four years, fewer children were included. In general, the yearly samples sizes were lower up to the age of five. This may be due to the increased difficulty of taking venous blood samples from very young children (no consent of parents/no blood samples available/insufficient amount of serum). As our study indicates that levels of certain serum lipids stabilize in early childhood already, further research focusing on these age groups is needed. Lastly, it must be noted that the composition of the LIFE Child study is slightly distorted with regards to the social class as measured by the Winkler index. Children from socially disadvantaged families are underrepresented [63]. Nonetheless, previous work based on the LIFE Child cohort has shown that in terms of prevalence of obesity and dyslipidemia as well as familial hypercholesterolemia, the LIFE Child cohort is representative for the German population [31].

## Conclusion

By analyzing serum lipid trajectories in healthy children aged 0.25 to 18 years on a year-to-year basis, our study complements already existing research. We showed *normal* serum levels to be stable over time. This was also true for *adverse* TC, LDL-C and apolipoprotein B levels. We found *adverse* serum lipid levels to be more and more stable with increasing age. Moreover, TC, LDL-C and apolipoprotein B were already stable in early childhood and subject to presumably puberty related changes. Our findings suggest that TC, LDL-C and apolipoprotein B might be of higher predictive value than HDL-C, TG or apolipoprotein A.

## Supplementary Information

**Additional file 1: Supplement 1.** Study population (overview). **Supplement 2.** Mean levels of serum lipids (TC, LDL, HDL, ApoA, ApoB, TG) by age group; *n* = total number of observations. **Supplement 3.** Mean prospective frequencies for children with normal, at-risk or adverse levels of TC. **Supplement 4.** Mean prospective frequencies for children with normal, at-risk or adverse levels of LDL. **Supplement 5.** Mean prospective frequencies for children with normal, at-risk or adverse levels of HDL. **Supplement 6.** Mean prospective frequencies for children with normal, at-risk or adverse levels of TG. **Supplement 7.** Mean prospective frequencies for children with normal, at-risk or adverse levels of ApoA. **Supplement 8.** Mean prospective frequencies for children with normal, at-risk or adverse levels of ApoB. **Supplement 9.** Comparison of mean prospective relative frequencies and r between entire study population and subset including the LIFE Child health cohort.

**Additional file 2: Figure 4.** TC risk profile trajectory (pRF) stratified by risk group. **Figure 5.** LDL-C risk profile trajectory (pRF) stratified by risk group. **Figure 6.** HDL-C risk profile trajectory (pRF) stratified by risk group. **Figure 7.** TG risk profile trajectory (pRF) stratified by risk group. **Figure 8.** Apolipoprotein A risk profile trajectory (pRF) stratified by risk group. **Figure 9.** Apolipoprotein B risk profile trajectory (pRF) stratified by risk group.

## Data Availability

The datasets analyzed during the current study are not publicly available. The LIFE Child study is a study collecting potentially sensitive information. Data cannot be shared publicly because there exist ethical and legal restrictions. Publishing data sets is not covered by the informed consent provided by the study participants. However, every researcher affiliated with a research institution can request data access. Researchers interested in accessing and analyzing data collected in the LIFE Child study may contact the data use and access committee (dm@life.uni-leipzig.de).
