# Supplementary material for: Tracking of serum lipids in healthy children on a year-to-year basis
Source: BMC Cardiovasc Disord. 2023 Aug 2;23:386. doi: 10.1186/s12872-023-03391-9 (PMC10398926; doi:10.1186/s12872-023-03391-9)

Figure 4: TC risk profile trajectory (pRF) stratified by risk group (online only)


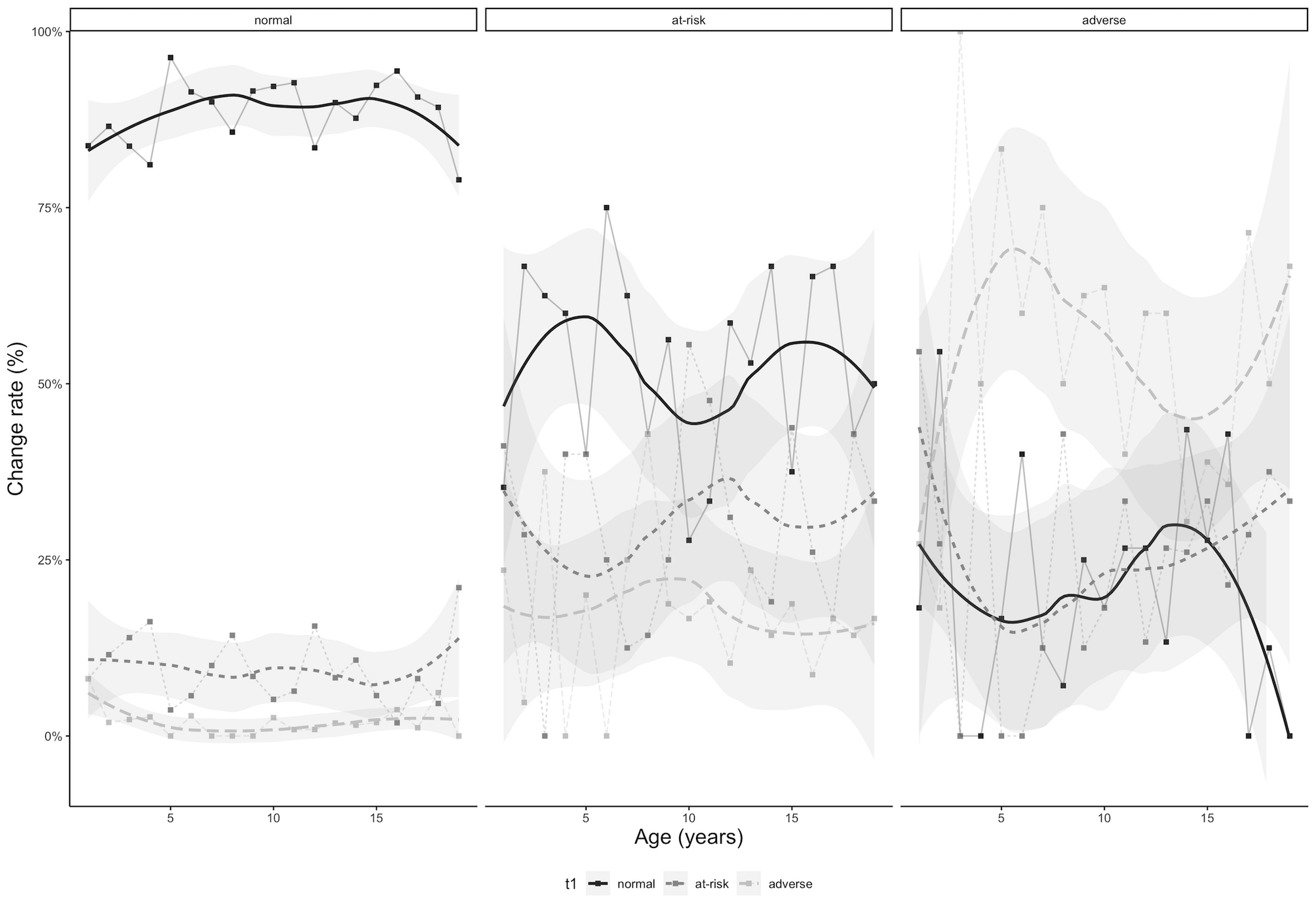


Figure 5: LDL-C risk profile trajectory (pRF) stratified by risk group (online only)


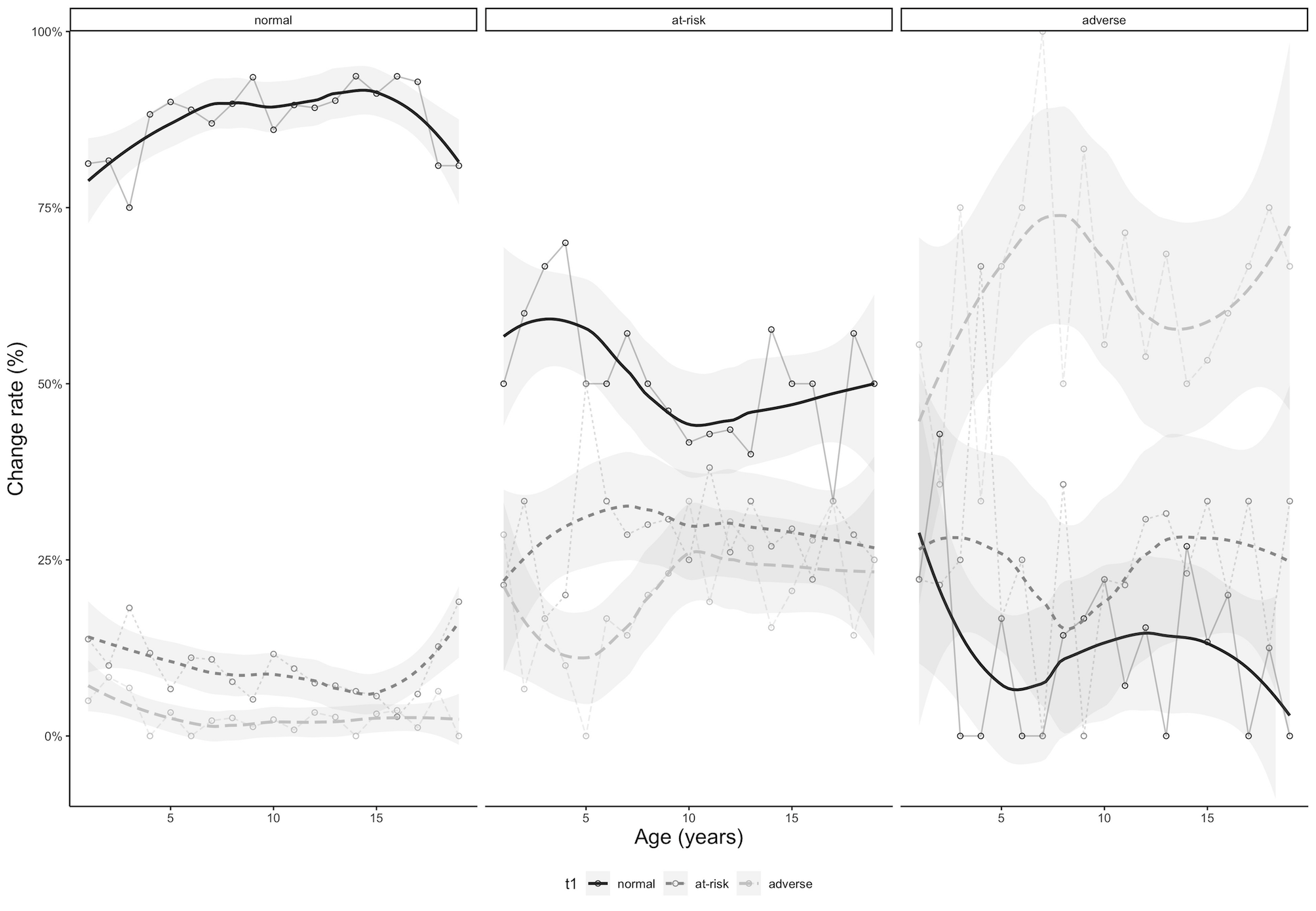


Figure 6: HDL-C risk profile trajectory (pRF) stratified by risk group (online only)


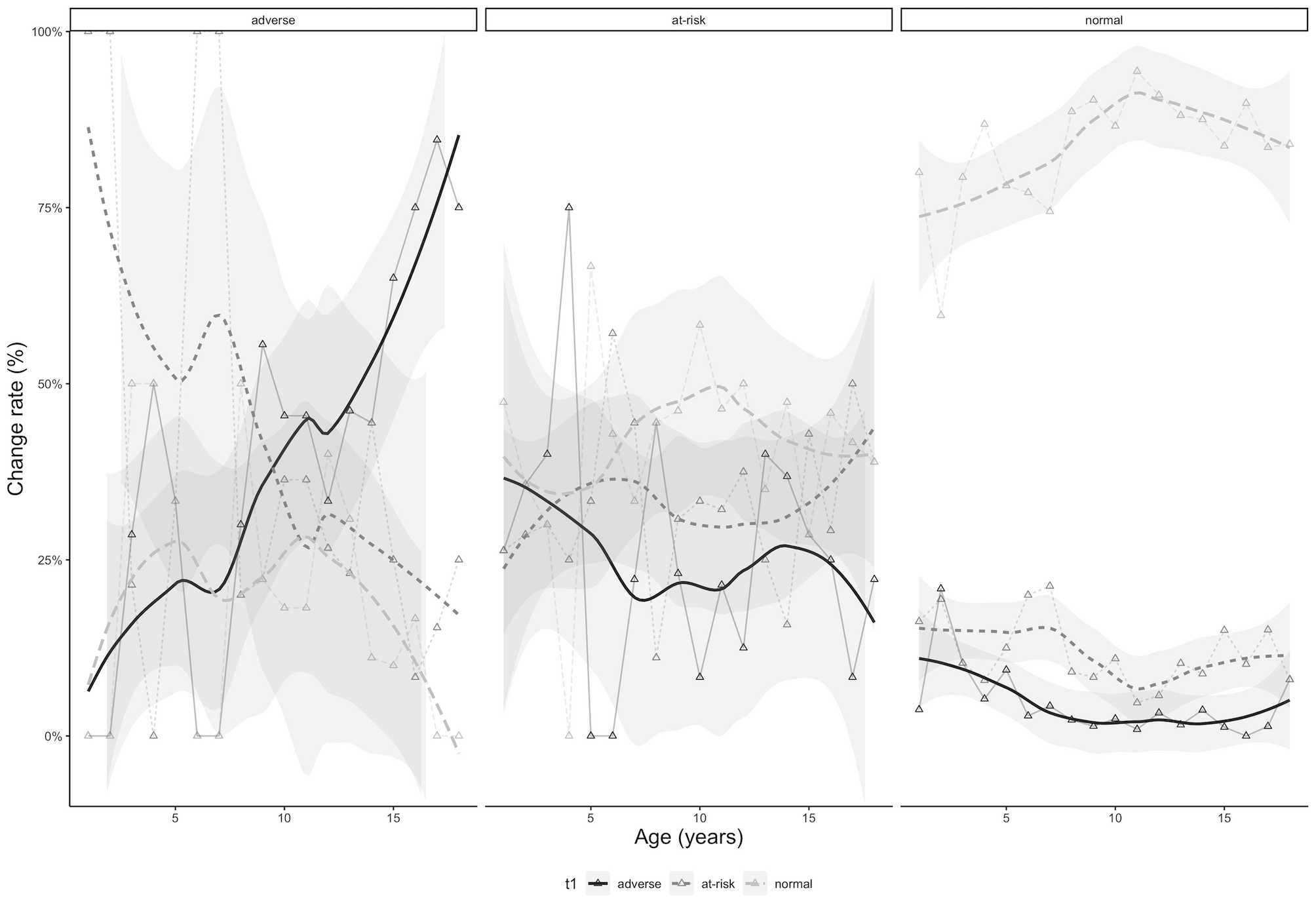


Figure 7: TG risk profile trajectory (pRF) stratified by risk group (online only)


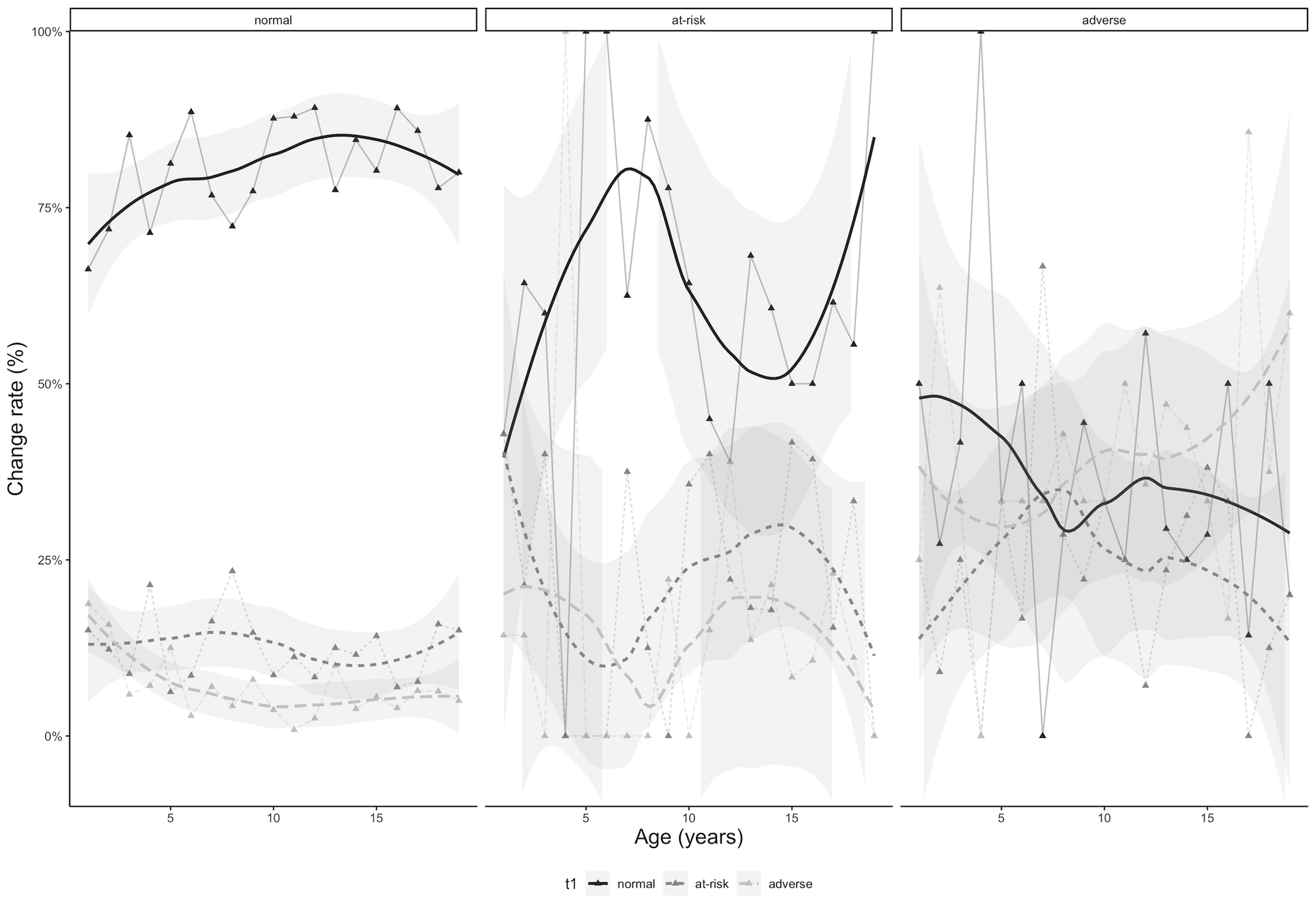


Figure 8: ApoA risk profile trajectory (pRF) stratified by risk group (online only)


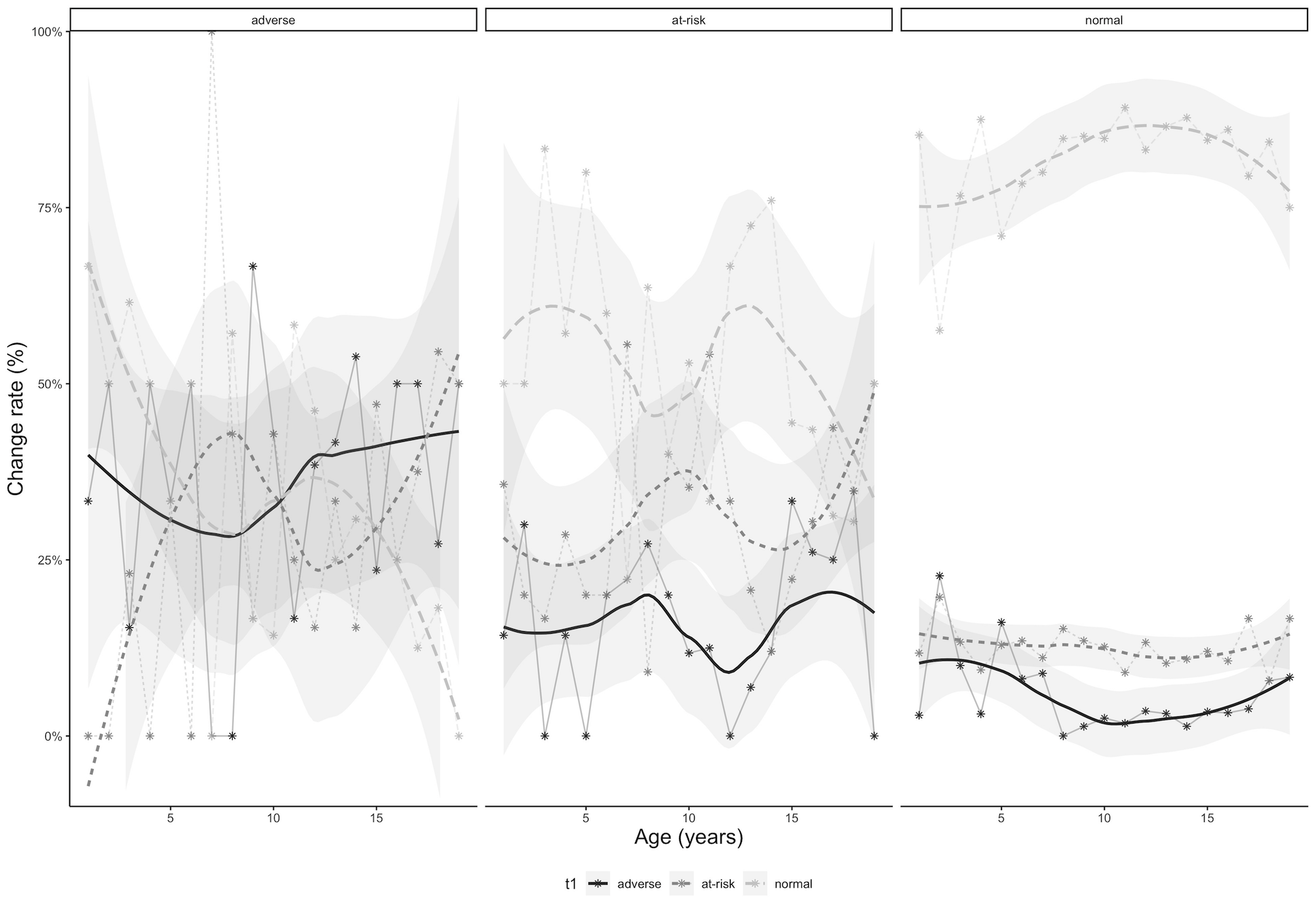


Figure 9: ApoB risk profile trajectory (pRF) stratified by risk group (online only)


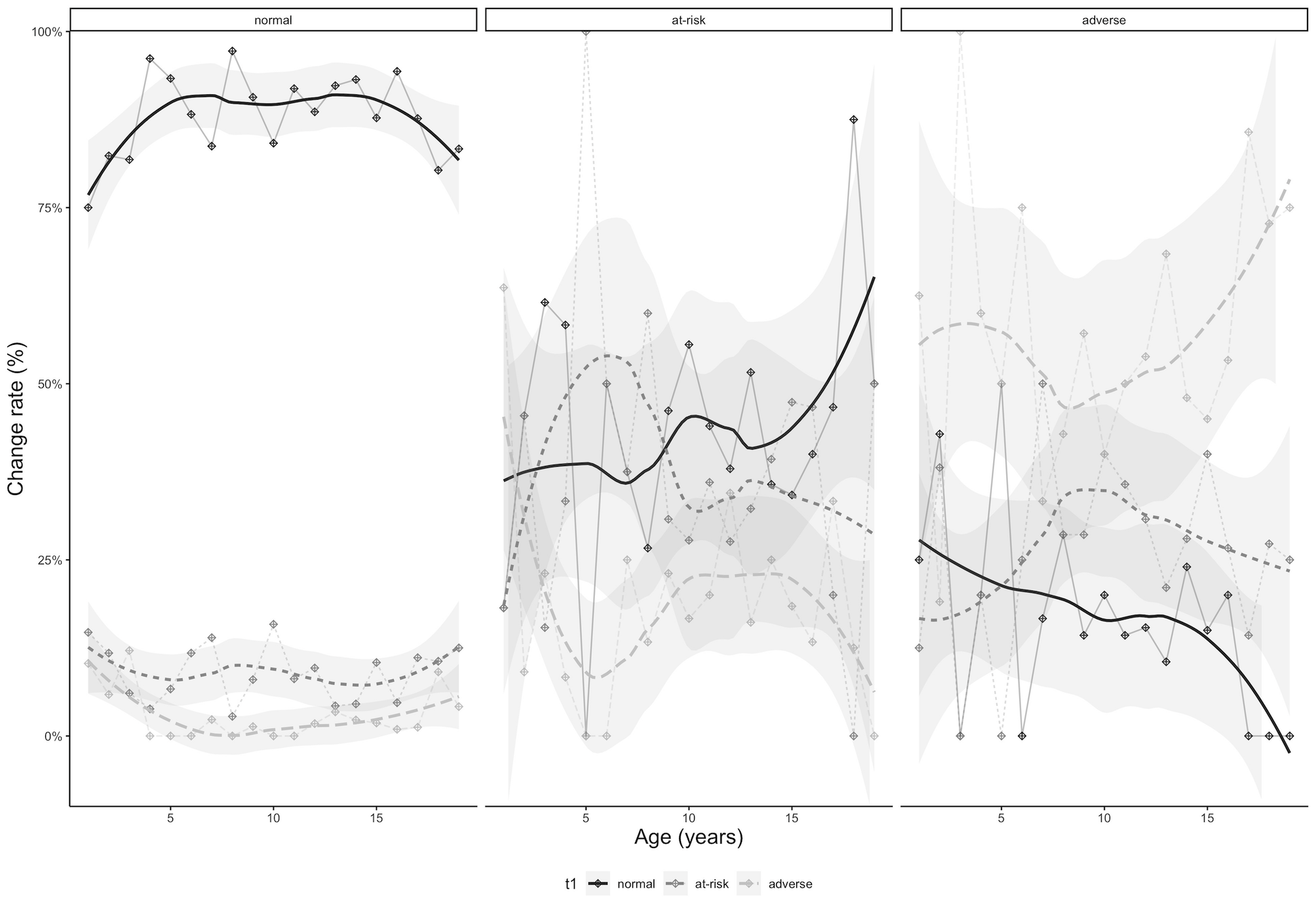

Supplement: Supplementary file 2 — Additional file 2: Figure 4. TC risk profile trajectory (pRF) stratified by risk group. Figure 5. LDL-C risk profile trajectory (pRF) stratified by risk group. Figure 6. HDL-C risk profile trajectory (pRF) stratified by risk group. Figure 7. TG risk profile trajectory (pRF) stratified by risk group. Figure 8. Apolipoprotein A risk profile trajectory (pRF) stratified by risk group. Figure 9. Apolipoprotein B risk profile trajectory (pRF) stratified by risk group. [file 12872_2023_3391_MOESM2_ESM.docx]
